# Outbreak of Imported Seventh Pandemic *Vibrio cholerae* O1 El Tor, Algeria, 2018

**DOI:** 10.3201/eid2806.212451

**Published:** 2022-06

**Authors:** Nabila Benamrouche, Chafika Belkader, Elisabeth Njamkepo, Sarah Sihem Zemam, Soraya Sadat, Karima Saighi, Dalila Torkia Boutabba, Faiza Mechouet, Rym Benhadj-Slimani, Fatma-Zohra Zmit, Jean Rauzier, Farid Kias, Souad Zouagui, Corinne Ruckly, Mohamed Yousfi, Amel Zertal, Ramdane Chouikrat, Marie-Laure Quilici, François-Xavier Weill

**Affiliations:** Institut Pasteur d’Algérie, Algiers, Algeria (N. Benamrouche, C. Belkader, S.S. Zemam, S. Sadat, D.T. Boutabba, R. Benhadj-Slimani, F. Kias);; University of Algiers I, Algiers (N. Benamrouche, F. Mechouet, F.-Z. Zmit, A. Zertal);; Institut Pasteur, Université Paris Cité, Paris, France (E. Njamkepo, J. Rauzier, C. Ruckly, M.-L. Quilici, F.-X. Weill);; Public Hospital Establishment of Boufarik, Blida, Algeria (K. Saighi, M. Yousfi, R. Chouikrat);; Specialized Hospital Establishment El Hadi Flici, Algiers (F. Mechouet, F.-Z. Zmit, A. Zertal);; University Hospital of Oran and University of Oran I, Oran, Algeria (S. Zouagui)

**Keywords:** cholera, Vibrio cholerae O1, bacteria, antimicrobial resistance, enteric infections, genomics, outbreak, Africa, Algeria

## Abstract

After a lull of >20 years, Algeria experienced a cholera outbreak in 2018 that included 291 suspected cases. We found that outbreak isolates were *Vibrio cholerae* O1 serotype Ogawa from seventh pandemic El Tor sublineage AFR14, which corresponds to a new introduction of cholera into Africa from South Asia.

Cholera, a life-threatening diarrheal disease, is caused by *Vibrio cholerae* O1, or more rarely O139, serogroup bacteria that produce cholera toxin (CTX) and induce rapid massive loss of body fluids (*1*). Cholera has been a serious public health problem since its introduction into Africa in 1970, during the seventh cholera pandemic (*2*). This pandemic, caused by the novel *V. cholerae* O1 lineage El Tor (seventh pandemic El Tor), began in Indonesia in 1961 (*2*,*3*). After 60 years, ≈2.9 million cholera cases and ≈95,000 deaths still occur annually (*4**,**5*). During 2009–2012, nearly 60% of global cholera cases and deaths occurred in sub-Saharan Africa, but North Africa was considered cholera-free (*5*).

Algeria is a large country (2,381,741 km^2^) in North Africa (*6*). The World Bank (https://www.worldbank.org) ranks Algeria as the third largest economy in the Middle East and North Africa region. In 2018, Algeria had ≈42.2 million inhabitants, ≈30.6 million of whom lived in urban areas (Macrotrends LLC, https://www.macrotrends.net). 

Algeria reported cholera cases to the World Health Organization from 1971 (1,332 cases, 110 deaths) through 1994 (118 cases, 4 deaths), with a peak in 1979 (2,513 cases, 94 deaths) (Global Health Observatory, https://www.who.int/data/gho) (Figure 1). After a lull of >20 years, on August 23, 2018, the country’s ministry of health announced a cholera outbreak in north Algeria (*7*). During August 7–September 27, 2018, Algeria reported 291 suspected cholera cases, including 270 persons who were hospitalized, in 7 wilayas (provinces): 6 in north-central Algeria (Bouira, Blida, Algiers, Tipaza, Aïn Defla, and Médéa) and 1 in northwest Algeria (Oran).

**Figure 1:**
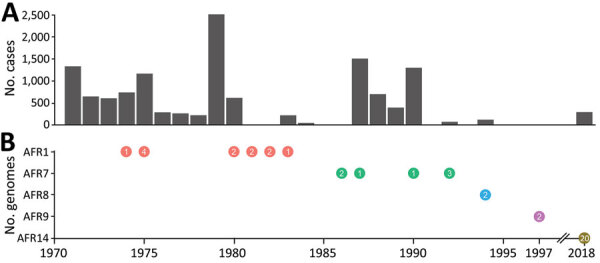
Cholera cases and seventh pandemic *Vibrio cholerae* O1 biotype El Tor sublineages, Algeria, 1971–2018. A) Number of cholera cases reported to the World Health Organization (WHO) by Algeria per year. For 2018, no cases were reported to WHO, but 291 suspected cases are indicated. B) Number of sequenced genomes detected from various sublineages per year of isolation. Orange circles indicate AFR1, green indicate AFR7, blue indicates AFR 8, purple AFR9, gold AFR14. Numbers in circles indicate the number of isolates.

We used conventional microbiology and whole-genome sequencing to characterize virulence and antimicrobial resistance of clinical and environmental isolates collected during this outbreak. We also performed a phylogenomic analysis of >1,200 seventh pandemic El Tor genomes to determine whether the 2018 outbreak in Algeria was caused by a sublineage previously circulating in the country, a sublineage circulating in sub-Saharan Africa, or a new sublineage imported from elsewhere.

## The Study

The Enterobacteria Laboratory of the Institut Pasteur d’Algérie performed microbial analyses for case confirmation (Appendix 1 Table 1; Appendix 2). During August 14–September 27, 2018, this laboratory received 695 stool samples from hospitals or hygiene laboratories in 7 wilayas, 277 from suspected case-patients and 418 from case-contacts, as well as 24 clinical isolates (14 from patients and 10 from case-contacts) and 5 environmental isolates (2 from wastewater, 2 from public drinking water sources, and 1 from stored water) for confirmation. In all, we confirmed 97/291 (33.3%; 95% CI 28.2%–38.9%) suspected cases as *V. cholerae* O1 El Tor serotype Ogawa carrying the *ctxA* gene and 29/428 (6.8%; 95% CI 4.8%–9.6%) case-contacts as asymptomatic carriers. Of the 5 environmental isolates, we also confirmed 2 from wastewater and 1 from stored water as serotype Ogawa.

All *V. cholerae* O1 isolates had the same antimicrobial resistance profile: resistance to streptomycin, sulfamethoxazole, trimethoprim, sulfamethoxazole/trimethoprim, nalidixic acid; decreased susceptibility to ciprofloxacin; and intermediate resistance to chloramphenicol and nitrofurantoin (Table). However, isolates were susceptible to doxycycline, azithromycin, β-lactams, and colistin.

**Table:** Characteristics of *Vibrio cholera* 01 epidemic strain, Algeria, 2018*

Category	Strain characteristic
Serogroup, serotype, biotype	O1, Ogawa, El Tor
Genomic wave	3
Sublineage	Seventh pandemic *V. cholerae *O1 biotype El Tor
Genetic markers	*ctxB7*, *tcpA*^CIRS101^, VSP-IIΔ‡
AMR profile, antimicrobial drug (MIC)†	
Streptomycin (64–128 mg/L)	Resistant
Sulfamethoxazole (1,024 mg/L)	Resistant
Trimethoprim/sulfamethoxazole (32 mg/L)	Resistant
Trimethoprim (32 mg/L)	Resistant
Chloramphenicol (16 mg/L)	Intermediate
Nalidixic acid (256 mg/L)	Resistant
Ciprofloxacin (0.25 mg/L)	Decreased susceptibility
Nitrofurantoin (64 mg/L)	Intermediate
Colistin (2 mg/L)	Susceptible
Horizontally acquired AMR elements	ICE*Vch*Ind5
Horizontally acquired AMR genes	*strAB*, *floR*, *sul2*, *dfrA1*
Chromosomal gene mutations, AMR phenotype	
* gyrA*_S83I and *parC*_S85L	Resistance to nalidixic acid; decreased susceptibility to ciprofloxacin
* nfsA*_R169C and *nfsB*_Q5Stop	Intermediate susceptibility to nitrofurantoin
* vprA*_D89N	Susceptibility to colistin

*Data were collected from 20 sequenced outbreak isolates. AMR, antimicrobial resistance; ICE*Vch*Ind5, integrative conjugative element of the SXT/R391 family; VSP-IIΔ, deletion in *Vibrio* seventh pandemic island II. †MICs according to Clinical and Laboratory Standards Institute (https://clsi.org/media/1450/m45ed3_sample.pdf). ‡Deletion encompassing VC_0495-0512 according to GenBank accession no. AE003852.

**Figure 2:**
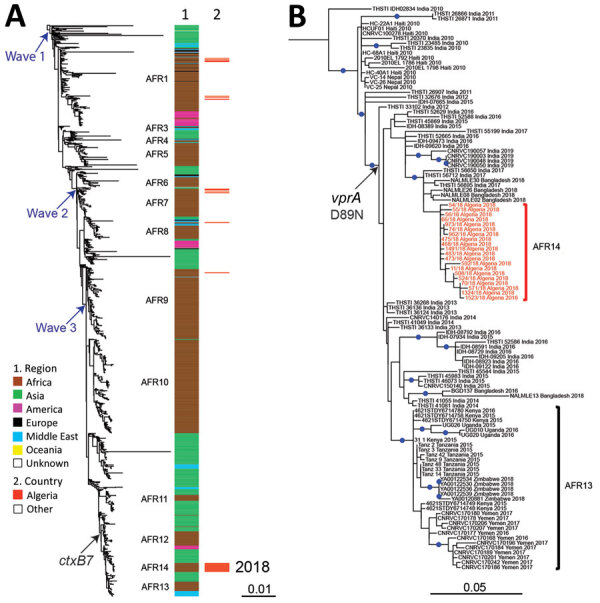
Phylogenomic analyses of *Vibrio cholerae* O1 El Tor isolates from Algeria, 2018. A) Maximum-likelihood phylogeny for 1,285 seventh pandemic *V. cholerae* biotype El Tor genomic sequences. A6 was used as the outgroup (Appendix 1, Table 4). Genomic waves and acquisition of *ctxB7* allele are indicated. Sublineages previously introduced into Africa (AFR1, AFR3–AFR13) are shown at the right of the tree. Column 1 indicates the geographic origins of the isolates; column 2 indicates isolates from the 2018 cholera outbreak in Algeria, all of which belong to a new seventh pandemic wave 3 sublineage AFR14. B) Maximum-likelihood phylogeny for 115 wave 3 *ctxB7* isolates belonging to the distal part of the tree in panel A. N16961 was used as the outgroup (Appendix 1, Table 4). The isolates belonging to AFR14 from the 2018 cholera outbreak in Algeria are shown in red. Acquisition of the polymyxin susceptibility–associated single nucleotide variant in *vprA* (D89N) is indicated. Blue dots indicate bootstrap values >90%. Scale bars indicate the number of nucleotide substitutions per variable site.

We used whole-genome sequencing, comparative genomics, and phylogenetic analysis to characterize a selection of 20 *V. cholerae* O1 isolates, 17 clinical and 3 environmental (Appendix 1 Tables 2, 3; Appendix 2). We placed these isolates in context with a global collection of 1,265 seventh pandemic El Tor genomic sequences (Appendix 1 Table 4), including 23 isolates collected in Algeria during 1971–1997. We constructed a maximum-likelihood phylogeny of 1,285 genomes with 10,339 single-nucleotide variants (SNVs) evenly distributed over the nonrepetitive, nonrecombinant core genome. All the isolates recovered in Algeria during 2018 belonged to the seventh pandemic El Tor lineage and clustered in the wave 3 clade containing isolates carrying the *ctxB7* allele (Figure 2, panel A) (*3*). The 2018 isolates did not belong to sublineages previously found in Algeria, including AFR1, which circulated during the 1970s and early 1980s; AFR7, which circulated during the mid- to late-1980s and early 1990s; or AFR8 and AFR9, which circulated during the mid-1990s (Figures 1, 2) (*8*). The 2018 isolates also did not belong to other sublineages found in Africa, including the most recently introduced AFR13 sublineage, previously known as T13 (*8**–**11*). AFR13 has been circulating in eastern Africa since 2015 and in Yemen since 2016 (Figure 1). A second phylogeny, restricted to 115 wave 3 *ctxB7* isolates from the distal part of the global tree, showed the 2018 isolates from Algeria are closely related to isolates from South Asia collected during 2017–2018 in India and Bangladesh (Figure 2, panel B). This finding suggests the 2018 cholera outbreak in Algeria was cause by a newly imported strain (sublineage AFR14) from South Asia, rather than resurgence of any sublineage previously in Algeria or introduction of a sublineage circulating elsewhere in Africa.

The median pairwise distance between the 20 isolates recovered during the 2018 outbreak was 2.5 (range 0–8) core-genome SNVs. All 20 isolates had similar genomic features (Table), including the toxin-coregulated pilus subunit A gene variant, *tcpA*^CIRS101^, a deletion (ΔVC_0495–0512) in the *Vibrio* seventh pandemic island II (VSP-II), and an SXT/R391 integrating conjugating element (ICE), called ICE*Vch*Ind5, encoding resistance to streptomycin (*strAB*), sulfonamides (*sul2*), trimethoprim (*dfrA1*), sulfamethoxazole/trimethoprim (*dfrA1* and *sul2*), and intermediate resistance to chloramphenicol (*floR*) (*8*). The Algeria isolates had mutations of *VC_0715*, resulting in the R169C substitution, and *VC_A0637*, resulting in the premature stop codon (Q5Stop) conferring intermediate nitrofuran resistance. Isolates also had mutations of the DNA gyrase, *gyrA* (S83I), and topoisomerase IV, *parC* (S85L), genes conferring resistance to nalidixic acid and decreased susceptibility to ciprofloxacin (*8*,*9*). In addition, isolates had a specific nonsynonymous SNV in the *vprA* gene (*VC_1320*), which resulted in the D89N substitution, conferring susceptibility to polymyxins (*9*), as reported for the AFR13 sublineage, although resistance to polymyxin B has been used as a marker of *V. cholerae* O1 biotype El Tor since the seventh pandemic began (*12*).

## Conclusions

The seventh pandemic El Tor wave 3 clade, containing isolates carrying the *ctxB7* allele, emerged in South Asia earlier this century (*9*,*13*) and has been exported from Asia >4 times: to West Africa in 2007 (AFR12 sublineage) (*8*), Haiti in 2010 (*14*), East Africa in 2013–2015 (AFR13) (*9*,*10*), and now North Africa (AFR14). Polymyxin-susceptible seventh pandemic El Tor isolates with a *vprA* mutation encoding the D89N substitution were identified in South Asia in 2012 (*15*), spread to Eastern Africa and Yemen (AFR13) (*9*,*10*), and then spread to Algeria (AFR14). 

Algeria controlled disease spread more swiftly in 2018 than during previous seventh pandemic El Tor introductions. The ministry of health led the epidemic response, initiated an emergency action plan at national and local levels, and enhanced epidemiologic surveillance and reporting. A health surveillance unit coordinated response actions and implemented recommendations. Designated hospitals managed suspected case-patients in isolation wards. Persons with suspected *V. cholerae* were hospitalized, isolated, rehydrated, and treated with doxycycline, erythromycin, azithromycin, ceftriaxone, or ciprofloxacin; patients were released only after a negative *V. cholerae* culture. Case-contacts were systematically screened, and asymptomatic carriers received chemoprophylaxis. In affected areas, the ministry of health reinforced bacteriologic monitoring of water sources, including drinking water, bore holes, wells, springs, and wadi (ravines that are dry except during rainy seasons), and took corrective action for sources with poor bacteriologic quality.

In summary, *V. cholerae* O1 isolates collected during a 2018 cholera outbreak in Algeria were a seventh pandemic El Tor sublineage, AFR14, newly introduced into Africa from South Asia. Our findings suggest that, in addition to appropriate control and prevention measures during outbreaks, such as those used in Algeria, reducing the burden of cholera in South Asia might aid in long-term control of cholera in Africa.

Appendix 1 Additional phenotype and genomic characteristics, antimicrobial resistance elements, and accession numbers of *Vibrio cholerae* O1 biotype El Tor isolates, Algeria, 2018.

Appendix 2 Additional methods used to investigate an outbreak of seventh pandemic *Vibrio cholerae* O1 biotype El Tor, Algeria, 2018.
