# Supplementary material for: Outbreak of Imported Seventh Pandemic Vibrio cholerae O1 El Tor, Algeria, 2018
Source: Emerg Infect Dis. 2022 Jun;28(6):1241–5. doi: 10.3201/eid2806.212451 (PMC9155889; doi:10.3201/eid2806.212451)
Supplement: Appendix 2 — Additional methods used to investigate an outbreak of seventh pandemic Vibrio cholerae O1 biotype El Tor, Algeria, 2018. [file 21-2451-Techapp-s2.pdf]

Article DOI: https://doi.org/10.3201/eid2806.212451

Outbreak of Imported Seventh Pandemic *Vibrio cholerae* O1 El Tor, Algeria, 2018

Appendix 2

Additional Methods

Culture, Identification, and Serotyping

The stool samples were analyzed for confirmation of the presence of *Vibrio cholerae* by standard culture methods (*1*), except for 10 samples collected from case-contacts for whom a Crystal VC cholera rapid diagnostic test (Arkray, https://www.arkray.co.in) was negative. The cultured bacterial strains were characterized by using the API 20 E identification system (bioMérieux, https://www.biomerieux.com), and serotyping (*1*).

Antimicrobial Susceptibility Testing

Antimicrobial susceptibility testing was determined by the disk diffusion method, on Mueller-Hinton agar, in accordance with Clinical and Laboratory Standards Institute (CLSI) guidelines (*2*). The following antimicrobial drugs were tested using Bio-Rad (https://www.bio-rad.com) cartridges: ampicillin (10 µg), amoxicillin/clavulanic acid (20/10 µg), piperacillin (100 µg), cefazolin (30 µg), cefoxitin (30 µg), cefotaxime (30 µg), ceftazidime (30 µg), cefepime (30 µg), imipenem (10 µg), meropenem (10 µg), nitrofurantoin (300 µg), streptomycin (10 µg), amikacin (30 µg), gentamicin (10 µg), chloramphenicol (30 µg), sulfamethoxazole (300 µg), trimethoprim (5 µg), trimethoprim-sulfamethoxazole (1.25/23.75 µg), tetracycline (30 µg), nalidixic acid (30 µg), ciprofloxacin (5 µg), azithromycin (15 µg), and colistin (10 µg). The MICs of amoxicillin/clavulanic acid, nitrofurantoin, streptomycin, chloramphenicol, sulfamethoxazole, trimethoprim, trimethoprim-sulfamethoxazole, tetracycline, nalidixic acid, and ciprofloxacin were determined with Etests (bioMérieux) on 8 isolates chosen on the basis of city of isolation. The MICs of ampicillin and colistin were determined on the same 8 isolates by the microdilution method, in accordance with CLSI guidelines (*2*).

CLSI interpretative criteria for the antibiotic susceptibility testing of *Vibrio* spp. (M45 document) were used when available (*2*). For trimethoprim, nitrofurantoin, nalidixic acid, ciprofloxacin, and colistin the interpretative criteria for Enterobacteriaceae/*Salmonella* spp. (M100-S30 document) were used (*3*). Interpretive criteria for streptomycin, not available in the M45 and M100 documents, were used as follow: susceptibility $\leq$16 mg/L and resistance $\geq$32 mg/L. Quality control for the antimicrobial susceptibility analysis was performed with *Escherichia coli* ATCC 25922 and *Pseudomonas aeruginosa* ATCC 27853 (for carbapenems), as recommended by CLSI guidelines (*2*).

Total DNA Extraction and Detection of the Cholera Toxin Gene by PCR

Total DNA was extracted with the Purelink Genomic DNA mini Kit (Thermo Fisher Scientific, https://www.thermofisher.com), in accordance with the manufacturer's recommendations.

A conventional PCR simplex assay for detection of the *ctxA* gene (primers: CTX2: 5′-CGGGCAGATTCTAGACCTCCTG-3′ and CTX3: 5′-CGATGATCTTGGAGCATTCCCAC-3′) was performed, as previously described (*4*).

Selection of *Vibrio cholerae* O1 Strains for Genome Sequencing

Twenty *V. cholerae* O1 isolates collected at different times, from different places, and sources during the 2018 outbreak were selected for study by the Pasteur Institute of Algeria. Their total DNA extracts were sent to the French National Reference Center for Vibrios and Cholera (FNRCVC), Institut Pasteur, Paris, France for genomic analyses. In addition, total DNA extracts from three *V. cholerae* O1 isolates collected in Algeria in 1992 were also sent to the FNRCVC.

Whole-Genome Sequencing

We analyzed 43 *V. cholerae* O1 biotype El Tor isolates from Algeria by whole-genome sequencing (Appendix 1 Table 4). Fourteen of these isolates collected between 1974 and 1997 were sequenced in a previous study (*5*). The 29 *V. cholerae* O1 biotype El Tor isolates sequenced here consisted of the 20 isolates from 2018, 2 from 1980, 2 from 1981, 3 from 1992, and 2 from 1997. The isolates from 1980, 1981, and 1997 were from the collection of the FNRCVC and

their total DNA was extracted with the Maxwell 16-cell DNA purification kit (Promega, https://www.promega.com) in accordance with the manufacturer's recommendations.

Whole-genome sequencing was carried out at the P2M sequencing platform of the Institut Pasteur, and at the genotyping and sequencing core facility of the Institut du Cerveau (Paris, France), on Illumina (https://www.illumina.com) platforms generating 150 bp paired-end reads, yielding a mean coverage of 276-fold (minimum 123-fold, maximum 389-fold).

Additional Genomic Data

Raw sequence files and assembled genomes from 1,257 7PET strains were downloaded from the European Nucleotide Archive (ENA) or GenBank databases and included in this study (Appendix 1 Table 4).

Genomic Sequence Analyses

The paired-end reads and draft or assembled genomes were mapped onto the reference genome of *Vibrio cholerae* O1 El Tor N16961, also known as A19 (GenBank accession nos. LT907989 and LT907990) with Snippy version 4.6.0/BWA version 0.7.17 (https://github.com/tseemann/snippy). Single-nucleotide variants (SNVs) were called with Snippy version 4.6.0/Freebayes version 1.3.2 under the following constraints: mapping quality of 60, a minimum base quality of 13, a minimum read coverage of 4, and a 75% read concordance at a locus for a variant to be reported. An alignment of core genome SNVs was produced in Snippy for phylogeny inference.

Short reads were assembled with SPAdes version 3.15.2 (*6*). The various genetic markers were analyzed with BLAST version 2.2.26 against reference sequences of the O1 *rfb* gene, *ctxB*, *wbeT*, and the whole locus of VSP-II, as previously described (*5*).

The presence and type of acquired antimicrobial resistance genes (ARGs) or ARG-containing structures were determined with ResFinder version 4.0.1 (https://cge.cbs.dtu.dk/services/ResFinder), BLAST analysis against GI-15, Tn*7*, and SXT/R391 integrative and conjugative elements, and PlasmidFinder version 2.1.1 (https://cge.cbs.dtu.dk/services/PlasmidFinder). The presence of mutations in the genes encoding resistance to quinolones (*gyrA*, *parC*), resistance to nitrofurans (*VC_0715* and *VC_A0637*), or

restoring susceptibility to polymyxin B (*vprA)* was investigated by manual analysis of the sequences assembled de novo with BLAST, as previously described (*5*,*7*).

Phylogenetic Analysis

Repetitive (insertion sequences and the TLC-RS1-CTX region) and recombinogenic (VSP-II) regions in the alignment were masked (*5*). Putative recombinogenic regions were detected and masked with Gubbins version 2.4.1 (*8*). A maximum-likelihood (ML) phylogenetic tree was built from an alignment of 10,339 chromosomal SNVs, with RAxML version 8.2.12, under the GTR model with 200 bootstraps (*9*). This global tree was rooted on the A6 genome, and visualized with Interactive Tree of Live (iTOL) version 5 (https://itol.embl.de) (*10*). A second phylogenetic analysis was performed with the same methodology on 115 wave 3 *ctxB7* isolates, including all 20 isolates from the 2018 Algerian outbreak, belonging to the distal part of the global tree. This ML tree was built from an alignment of 506 chromosomal SNVs, rooted on the N16961 genome, and visualized with iTOL version 5.

Data Availability

Short-read sequence data were submitted to the ENA database (http://www.ebi.ac.uk/ena), under study accession number PRJEB48258 and their accession numbers are provided in Appendix 1 Table 4.

References

1. Centers for Disease Control and Prevention. Laboratory methods for the diagnosis of *Vibrio cholerae* Atlanta: The Centers; 1994. https://www.cdc.gov/cholera/pdf/laboratory-methods-for-the-diagnosis-of-vibrio-cholerae-chapter-6.pdf

2. Clinical and Laboratory Standards Institute. Methods for antimicrobial dilution and disk susceptibility testing of infrequently isolated or fastidious bacteria; approved guideline. 3rd edition (CLSI guidelines M45). Wayne (PA): the Institute; 2016.

3. Clinical and Laboratory Standards Institute. Performance standards for antimicrobial susceptibility testing. 30th edition. CLSI supplement M100. Wayne (PA): the Institute; 2020.

4. Fields PI, Popovic T, Wachsmuth K, Olsvik O. Use of polymerase chain reaction for detection of toxigenic *Vibrio cholerae* O1 strains from the Latin American cholera epidemic. J Clin Microbiol. 1992;30:2118–21. PubMed https://doi.org/10.1128/jcm.30.8.2118-2121.1992

5. Weill FX, Domman D, Njamkepo E, Tarr C, Rauzier J, Fawal N, et al. Genomic history of the seventh pandemic of cholera in Africa. Science. 2017;358:785–9. PubMed https://doi.org/10.1126/science.aad5901

6. Bankevich A, Nurk S, Antipov D, Gurevich AA, Dvorkin M, Kulikov AS, et al. SPAdes: a new genome assembly algorithm and its applications to single-cell sequencing. J Comput Biol. 2012;19:455–77. PubMed https://doi.org/10.1089/cmb.2012.0021

7. Weill FX, Domman D, Njamkepo E, Almesbahi AA, Naji M, Nasher SS, et al. Genomic insights into the 2016–2017 cholera epidemic in Yemen. Nature. 2019;565:230–3. PubMed https://doi.org/10.1038/s41586-018-0818-3

8. Croucher NJ, Page AJ, Connor TR, Delaney AJ, Keane JA, Bentley SD, et al. Rapid phylogenetic analysis of large samples of recombinant bacterial whole genome sequences using Gubbins. Nucleic Acids Res. 2015;43:e15. PubMed https://doi.org/10.1093/nar/gku1196

9. Stamatakis A. RAxML-VI-HPC: maximum likelihood-based phylogenetic analyses with thousands of taxa and mixed models. Bioinformatics. 2006;22:2688–90. PubMed https://doi.org/10.1093/bioinformatics/btl446

10. Letunic I, Bork P. Interactive Tree Of Life (iTOL) v4: recent updates and new developments. Nucleic Acids Res. 2019;47(W1):W256–9. PubMed https://doi.org/10.1093/nar/gkz239
